# Indication for mild therapeutic hypothermia based on an initial Glasgow Coma Scale motor score

**DOI:** 10.1186/s40560-015-0110-7

**Published:** 2015-10-31

**Authors:** Toru Hifumi, Kenya Kawakita, Tomoya Okazaki, Satoshi Egawa, Yutaka Kondo, Tomoaki Natsukawa, Hirotaka Sawano

**Affiliations:** Emergency Medical Center, Kagawa University Hospital, 1750-1 Ikenobe Miki, Kita, Kagawa 761-0793 Japan; Department of Surgery, Beth Israel Deaconess Medical Center, Harvard Medical School, 330 Brookline Avenue, Boston, MA 02215 USA; Senri Critical Care Medical Center, Osaka Saiseikai Senri Hospital, 1-1-6 Tsukumodai, Suita city, Osaka Japan

## Abstract

Although neurological evaluation using the Glasgow Coma Scale motor score is mandatory for post-cardiac arrest patients, further study is required to determine if this score can be used as an indicator for mild therapeutic hypothermia. Although the current study conducted by Natsukawa et al. presents interesting data, there are some critical issues regarding study design, selection bias, and interpretation of study results that should be pointed out.

## Correspondence

### Letter to the editor

We read with great interest the article titled “At what level of unconsciousness is mild therapeutic hypothermia indicated for out-of-hospital cardiac arrest: a retrospective, historical cohort study” by Natsukawa et al. [1]. However, there are some critical issues in this study that should be pointed out.

First, we could not identify the data that directly support the authors’ conclusion that mild therapeutic hypothermia (MTH) may be unnecessary in patients with a Glasgow Coma Scale (GCS) motor response score of 5 or higher. In Table three, the authors suggested that not only a GCS motor response score of 5 or 6 but also MTH were the independent factors predictive of good neurological outcomes in all study populations. Therefore, MTH should be considered in all study patients, including those with GCS motor scores of 5 or 6.

Second, the authors combined patients with GCS motor scores of 5 and 6 before analyzing the data and drawing conclusions. However, GCS motor scores of 5 and 6 should not be combined into the same category because we know that, in clinical practice, patients with GCS motor score 6 are clearly different from those with GCS motor scores 1–5.

Third, because this study is a single-center retrospective study, selection bias should be discussed. We recently published a study on the impact of the GCS motor score at admission on neurological outcomes in out-of-hospital cardiac arrest patients receiving therapeutic hypothermia [2] In our study, 25 % (1/4) of patients with GCS motor score 5 had a poor neurological outcome despite MTH; however, 52 % (130/249) of patients with an initial GCS motor score of 1 who underwent MTH had good neurological outcomes. However, in the study by Natsukawa et al., patients with GCS motor score 1 who underwent MTH had extremely poor outcome [favorable neurological outcome; 7/53 (13.2 %) (Table two)]. This was considered to be due to the existence of patients with cardiopulmonary arrest of non-cardiac origin. Therefore, this study group contained a more heterogeneous population, which makes it more difficult to interpret the study results.

Fourth, we should not rely on a single parameter, i.e., the GCS motor score in this case, to determine the indications for MTH because other physical examination findings, such as the pupil size, are also associated with the neurological outcome in patients who undergo MTH [2]. Based on the current evidence, an initial GCS motor score can provide at least some baseline objective data for prognosis discussions with surrogate decision makers [2].

In conclusion, although neurological evaluation using the GCS motor score is mandatory for post-cardiac arrest patients, further study is required to determine if this score can be used as an indicator for mild therapeutic hypothermia.

### Response

We appreciate Dr. Hifumi’s well-advised comments about our paper [1] “At what level of unconsciousness is mild therapeutic hypothermia indicated for out-of-hospital cardiac arrest: a retrospective, historical cohort study.”

**Fig. 1:**
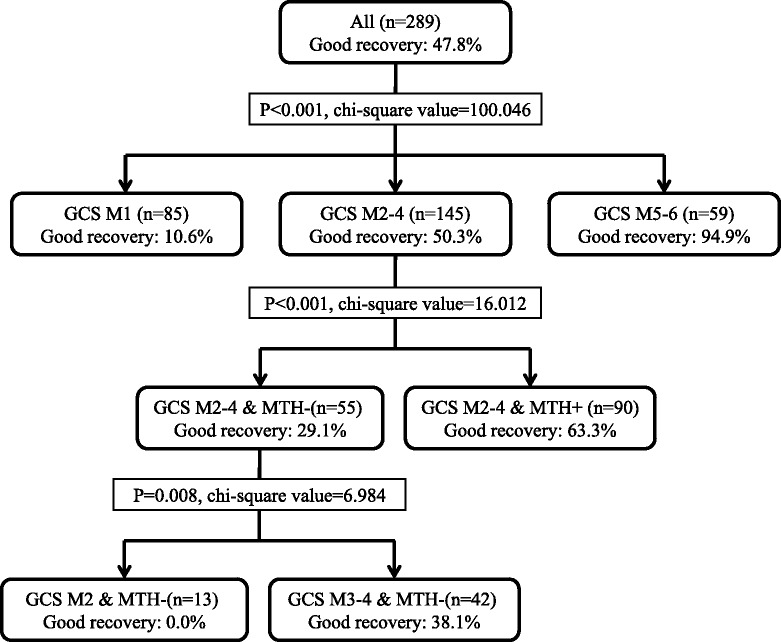
Chi-squared automatic interaction detection classification tree for good recovery at 30 days after hospital admission. GCS M1: patients classified with a GCS motor response score of 1. GCS M2–4: patients classified with a GCS motor response score from 2 to 4. GCS M5–6: patients classified with a GCS motor response score of 5 or higher. GCS M2–4 and MTH+: patients classified with a GCS motor response score from 2 to 4 and treated with MTH. GCS M2–4 and MTH−: patients classified with a GCS motor response score from 2 to 4 and treated without MTH. GCS M2 and MTH−: patients classified with a GCS motor response score of 1 and treated without MTH. GCS M3–4 and MTH−: patients classified with a GCS motor response score from 3 to 4 and treated without MTH

We will now outline the reasons why we concluded that mild therapeutic hypothermia (MTH) may be unnecessary in patients with a Glasgow Coma Scale (GCS) motor response score of 5 or higher, although MTH was an independent predictive factor for good neurological outcome, and the reason that we combined patients with GCS motor response score of 5 and 6. Firstly, we performed chi-squared automatic interaction detection (CHAID) analysis with GCS motor response score and MTH as independent variables and good recovery at 30 days after admission as the dependent variable. The tree created after applying CHAID is shown in Fig. 1. The terminal branches of the tree represent CHAID-derived homogeneous categories (terminal nodes). We obtained five terminal nodes. Patients classified with a GCS motor response score of 5 or higher had the highest percentage of good recovery. Secondly, the percentage of patients with a good recovery in the GCS M5 and M6 groups was around 100 %, and for the patients with a bad recovery in the GCS M5 and M6 groups, it was difficult to believe that implementing MTH would have improved the CPC at 30 days after hospital admission.

The primary reason that the percentage of good recovery of patients with GCS motor response score 1 in our study (7/53, 13.2%) was lower than that in the study by Hifumi et al. [2] (130/249, 52 %) is that our study population included more severe patients, such as patients who underwent E-CPR and patients with non-cardiac cause for cardiac arrest such as hypoxia and hypovolemia. The study by Hifumi et al. did not include patients who underwent E-CPR or patients with non-cardiac cause for cardiac arrest.

Regarding why the percentage of bad recovery for patients with GCS motor response score 5 in our study (1/32, 3.2 %) was lower than those in the study by Hifumi et al. (1/4, 25 %), there are three reasons. Firstly, because the number of patients with a GCS motor response score of 5 in the study by Hifumi et al. was only four, a small number, we cannot discuss whether the percentage is high or low, and on the other hand, we also regard the number of patients with bad recovery in the study by Hifumi et al., one, as a small number. In our study, one case with GCS motor response score 5 with Cerebral Performance Category 5 that had not undergone MTH was admitted to the hospital due to malnutrition and died in hospital from an inability to control the primary disease. In this case, it was difficult to believe that MTH would have improved the CPC at 30 days after hospital admission. Secondly, in the study by Hifumi et al., there were multiple different hospital centers each with different inclusion criteria, a different protocol of MTH, and a different capacity of the intensive care unit. These factors resulted in variation in results between sites and a relatively high percentage of bad recovery in patients with GCS motor response score 5. Thirdly, the study by Hifumi et al. included only patients who were treated with MTH. There was no comparison to patients who were treated without MTH, and it is possible that there was no difference in the percentage of good recovery between patients who were treated with MTH and patients who were treated without MTH.

Regarding the question about relying on a single parameter, the GCS M score, to determine the indication for MTH, we must consider the significant adverse effects of MTH such as cardiac output insufficiency and coagulation disorder. Cardiogenic shock is common in post-cardiac arrest patients, and MTH can potentially worsen the situation. Also, post-cardiac arrest patients sometimes have thoracic trauma from prolonged chest compressions, and coagulation disorders could worsen any hemorrhage. We believe that it is better to treat with MTH only when the benefits of MTH outweigh the risks. We want to emphasize that we must prevent hyperthermia for all post-cardiac arrest patients admitted to ICU, however MTH may be unnecessary for patients with a GCS motor response score of 5 or higher.

Regarding the requirement for further study, we also wrote in our article that we propose that a prospective study to evaluate the neurological outcome of GCS motor response score 5 with or without MTH in post-cardiac arrest patients would be beneficial.

## Abbreviations

GCS: Glasgow Coma Scale
MTH: mild therapeutic hypothermia
